# Effect of Aromatherapy on Dental Anxiety Among Orthodontic Patients: A Randomized Controlled Trial

**DOI:** 10.7759/cureus.5306

**Published:** 2019-08-02

**Authors:** Premkumar K S, Syed Aafaque, Sumalatha S, Narendran N

**Affiliations:** 1 Department of Orthodontics and Dentofacial Orthopaedics, Best Dental Science College and Hospital, Madurai, IND

**Keywords:** aroma therapy, orthodontics, lavender oil, rose oil, dental anxiety

## Abstract

Introduction: Dental anxiety is a distressing challenge faced by both the patients and the dental care provider, particularly in the Department of Orthodontics. The goal of this study was to assess the effect of aromatherapy (oils) on dental anxiety level among orthodontic patients and to compare the effect on anxiety levels between lavender oil, rose oil, and a placebo.

Materials and methods: A total of 72 patients (36 men and 36 women) who reported to the Department of Orthodontics were selected and randomly allocated into three groups: lavender oil, rose oil, and placebo. The ambient odor of those oils or placebo was maintained with a candle warmer, and the patients were made to wait in their respective rooms for 15 minutes. Participants' heart rate and blood pressure, being reliable and objective indicators of anxiety, were measured using pulse oximeter and sphygmomanometer, respectively. Additionally, a questionnaire, comprising demographic data and questions representing a modified Dental Anxiety Scale, was given to the patients to measure subjective anxiety levels before and after aromatic therapy. Paired t-test, analysis of variance, and the Wilcoxon signed-rank test were used for both subjective and objective analyses.

Results: Lavender oil and rose oil produced a significant reduction of dental anxiety level among orthodontic patients in both objective and subjective method (P ≤ .05); lavender oil demonstrated a greater significance in reducing the anxiety level when compared to rose oil.

Conclusion: Our findings indicate that aromatherapy offers promising effect against dental anxiety among orthodontic patients. Aromatherapy can be recommended as an easy alternative to reduce anxiety in patients before orthodontic treatment.

## Introduction

Anxiety is an emotion experienced by most individuals during their lifetime. Dental anxiety is anxiety that is associated with general dental care, the anticipation of treatment, fear of the unknown, fear of pain, or the relationship with dental professionals in the dental office. It is the most common psychological condition reported in dentistry, affecting a significant percentage of the population. Dental anxiety can be described as mild apprehension in response to an uncertain situation. It is of greater significance to dentists [1]. Some studies conclude that orthodontic patients are more anxious about treatment.

The presence of dental anxiety has important implications for both patients and clinicians. It can be problematic for clinicians because anxious patients can be difficult to manage and treat, or might delay or avoid their appointments [2,3]. From the patient’s perspective, those with dental anxiety tend to overestimate the experienced pain, report less satisfaction, have an aversion to future treatment, and reduced compliance [4-6]. All of these apply to orthodontic patients.

Anxiety is managed with either pharmacological or nonpharmacological methods. A common method of pharmacologic management of anxiety is through conscious sedation or general anesthesia [7]. This method is associated with some risks, requires additional equipment, cannot be applied to patients with allergies to other medications, and has many side effects [8].

A search for an alternative method focused on Complementary and Alternative Medicine (CAM) [9]. CAMs have long been regarded as popular nonpharmacological strategies for the treatment of anxiety. One CAM involves the use of aromatic oils to excite the olfactory system and reduce anxiety symptoms. Aromatherapy has claimed to be beneficial for mental, psychologic, and social aspects. Also, it is relatively free of adverse effects [10].

Lavender (*Lavandula angustifolia* and *Lavandula stoechas*) belongs to the Labiatae family and is well-known for its anxiolytic, relaxing aroma. Linalool, a sedative affecting gamma‑aminobutyric acid receptors in the central nervous system, and linalyl acetate, a narcotic, are the active ingredients in the plant [11]. The study conducted by Re et al. confirmed the significant effects of lavender on anxiety, while few studies have evaluated the effect of lavender on dental anxiety [12].

Rose (*Rosa rubiginosa*) oil has been shown to reduce anxiety during dental appointments. The current study aimed to assess the effect of aromatherapy on dental anxiety levels among orthodontic patients and to compare the effect of aromatherapy using lavender oil versus rose oil on anxiety levels among orthodontic patients.

## Materials and methods

The present study was a randomized controlled trial with parallel arms involving patients of 15 to 30 years of age reporting to the department of orthodontics at a dental college. The trial was double-blinded, the principal examiner and the statistician were blinded. Ethical clearance was obtained from the Institutional Ethical Clearance board (IRB/IEC Reference No: 2018-STF-BrV-KSP-16).

Participants were selected based on inclusion and exclusion criteria. The inclusion criteria were those with good general health and of age 15 to 35 years old. Subjects with breathing disorders and who were allergic to any of the ingredients of aromatherapy were excluded from the study. After receiving written consent from the subjects, the subjects were randomly divided into three groups: Group I was lavender, Group II was rose oil, and group III was placebo. Randomization was carried out using a simple lottery method to produce a random sample. This ensured that a uniform allocation of participants in the three groups.

The sample size was calculated with an Alpha error at the 5% significance level and the beta error at 80%. The target sample size calculated for each group was 21. Considering a 10% attrition, the sample size was calculated as 24 in each group. The final sample size was 72.

Methods of measuring anxiety

The anxiety was measured by objective and subjective methods.

Objective Measures

The psychophysiological responses produced by anxiety are associated with an increase in the activity of the sympathetic branch of the autonomic nervous system. Heart rate and blood pressure, being the reliable indicators of anxiety, were measured using a fingertip pulse oximeter and sphygmomanometer, respectively [13].

Subjective Measures 

A questionnaire was used to evaluate the subjective factors of assessing the effect of aromatherapy on dental anxiety. The questionnaire was developed to assess anxiety towards orthodontic treatment. A modified version of the Corah Dental Anxiety Scale that was pilot tested by the examiner prior to the study to ensure face and content validity of the questionnaire was used. The Modified Dental Anxiety Scale is commonly used to assess dental anxiety associated with the anticipation of treatment and specific dental procedures, bracket placing, drilling, and having an extraction of teeth. It consists of five questions with a standardized answer scheme for each question ranging from one (not anxious) to five (extremely anxious). A total score equal to seven or higher than 13 identifies an individual who is dentally anxious, with extremely anxious patients scoring 19 or higher. The scale’s reliability and validity were confirmed by psychometric testing.

Procedure

The questionnaire was distributed to those patients who reported to the outpatient department. The questionnaire was completed by the patients and returned to the principal examiner. The diffusive odor of lavender oil was sustained through a candle warmer in the waiting room of the patients. The candle warmer has upper and lower compartment; the lower compartment consists of an electrically operated light, the upper compartment contains the study material, water, lavender oil, or rose oil, as appropriate. The oil slowly diffused via the candle warmer and produced aroma. The same candle warmer was used in all groups for the purpose of standardization. However, lavender oil or rose oil was replaced by plain water in the placebo group. Patients were asked to complete the questionnaire after waiting for 15 minutes in their respective waiting rooms.

Statistical analysis

Collected data were analyzed using IBM SPSS Statistics for Windows, Version 23.0. (IBM Corp., Armonk, NY). The Wilcoxon signed-rank test, paired t-test, and analysis of variance were used for data analysis. The level of significance was set at .05.

## Results

In the present study, a total of 74 patients were included and were divided into three groups. An equal distribution (50%) of the study subjects based on gender was considered (Table 1). The distribution of the study subjects according to the age showed that the mean age was 20 years old, and the majority of the study subjects (n=18) were 21 years old (Table 2).

**Table 1:** Gender-wise distribution of the study subjects

GENDER	FREQUENCY	PERCENTAGE
Men	36	50%
Women	36	50%

**Table 2:** Age-wise distribution of the study subjects

AGE (years)	Frequency	Percent (%)	Mean	SD
15	6	8.3	20.5417	2.25137
18	6	8.3		
19	6	8.3		
20	9	12.5		
21	18	25.0		
22	12	16.7		
23	15	20.8		
Total	72	100.0		

The objective measurements (i.e., blood pressure and heart rate) showed that a significant reduction was seen only in the study groups, not in the control group (P < .05; Table 3).

**Table 3:** Intragroup comparison of blood pressure and heart rate pre- and post-aromatherapy ** - statistically significant P<0.05. BP - Blood pressure; HR- Heart rate.

Group	Variables	Paired Differences				Sig. (2-tailed)
		Mean	SD	95% Confidence Interval of the Difference		
				Lower	Upper	
LAVENDER OIL	PRESYSTOLIC BP (mmHg)	121.71	7.805	1.658	5.258	.001**
	POSTSYSTOLIC BP (mmHg)	118.25	9.218			
	PRE DIASTOLIC BP (mmHg)	84.21	5.192	.781	4.635	.008**
	POST DIASTOLIC BP (mmHg)	81.50	6.255			
	PRE HR (Beats/minute)	93.46	17.136	2.097	11.069	.006**
	POST HR (Beats/minute)	86.88	15.823			
ROSE OIL	PRESYSTOLIC BP (mmHg)	123.13	4.665	.152	2.014	.025**
	POSTSYSTOLIC BP (mmHg)	122.04	4.038			
	PRE DIASTOLIC BP (mmHg)	89.17	13.044	.821	.219	.029**
	POST DIASTOLIC BP (mmHg)	87.25	11.368			
	PRE HR (Beats/minute)	86.42	12.050	.380	.130	.024**
	POST HR (Beats/minute)	85.50	11.913			
PLACEBO	PRESYSTOLIC BP (mmHg)	120.71	5.552	.615	-1.314	.947
	POSTSYSTOLIC BP (mmHg)	120.75	5.135			
	PRE DIASTOLIC BP (mmHg)	89.17	13.044	.139	-.412	.377
	POST DIASTOLIC BP (mmHg)	89.29	12.987			
	PRE HR (Beats/minute)	86.42	12.050	.221	-.165	.200
	POST HR (Beats/minute)	86.13	12.127			

Intergroup comparison of pre- and post-aromatherapy systolic blood pressure showed no significant reduction (P = .131; i.e., the aromatherapies showed a similar reduction in systolic blood pressure to placebo). The higher reduction was seen in the lavender oil group, followed by the rose oil group (Table 4).

**Table 4:** Intergroup comparison systolic blood pressure in all the three groups ** - statistically significant P<0.05. BP - Blood pressure;

Variable: Systolic BP	Group	Paired Differences				N	Sig. (2-tailed)
		Mean mmHg	SD mmHg	95% Confidence Interval of the Difference			
				Lower mmHg	Upper mmHg		
BEFORE THERAPY	LAVENDER OIL	121.71	7.805	118.41	125.00	24	.397
	ROSE OIL	123.13	4.665	121.15	125.10		
	PLACEBO	120.71	5.552	118.36	123.05	24	
AFTER THERAPY	LAVENDER OIL	118.25	9.218	114.36	122.14		.131
	ROSE OIL	122.04	4.038	120.34	123.75	24	
	PLACEBO	120.75	5.135	118.58	122.92		

The mean diastolic blood pressure reduction following lavender oil rose oil, and placebo therapy among the study groups was significant (P = .036 ). A higher reduction was observed in the lavender oil group, followed by the rose oil group (Table 5).

**Table 5:** Intergroup comparison diastolic blood pressure in all the three groups ** - statistically significant P<0.05. BP - Blood pressure;

Variable: Diastolic BP	Group	Paired Differences				N	Sig. (2-tailed)
		Mean mmHg	SD mmHg	95% Confidence Interval of the Difference			
				Lower mmHg	Upper mmHg		
BEFORE THERAPY	LAVENDER OIL	84.21	5.192	81.25	89.35	24	.208
	ROSE OIL	89.17	13.044	79.98	97.02	24	
	PLACEBO	89.17	13.044	77.30	85.70	24	
AFTER THERAPY	LAVENDER OIL	81.50	6.255	78.86	84.14	24	.036**
	ROSE OIL	87.25	11.368	82.45	92.05	24	
	PLACEBO	89.29	12.987	83.81	94.78	24	

Intergroup comparison of the heart rate in pre- and post-aromatherapy showed no significant reduction (P = .939) i.e., the aromatherapies showed a similar reduction of systolic blood pressure to placebo (Table 6).

**Table 6:** Intergroup comparison of heart rate in all the three groups ** - statistically significant P<0.05. HR - Heart rate.

Variable: Heart rate	Group	Paired Differences				N	Sig. (2-tailed)
		Mean Beats/minute	SD Beats/minute	95% Confidence Interval of the Difference			
				Lower Beats/minute	Upper Beats/minute		
BEFORE THERAPY	LAVENDER OIL	93.46	17.136	86.22	100.69	24	.138
	ROSE OIL	86.42	12.050	81.33	91.51	24	
	PLACEBO	86.42	12.050	81.33	91.51	24	
AFTER THERAPY	LAVENDER OIL	86.88	15.823	80.19	93.56	24	.939
	ROSE OIL	85.50	11.913	80.47	90.53	24	
	PLACEBO	86.13	12.127	81.00	91.25	24	

The higher reduction was seen in the lavender oil group, followed by the rose oil group. The intergroup comparison of the subjective data via Wilcoxon signed-rank test reveals that there was a significant difference seen in all five questions (P < .05; Table 7).

**Table 7:** Intragroup comparison of the pre- and post-test questionnaire (Wilcoxon signed-rank test) ** - statistically significant P<0.05.

QUESTIONS	LAVENDER OIL			ROSE OIL			PLACEBO		
	Mean	SD	P	Mean	SD	P	Mean	SD	P
PREQUES1	4.0833	.58359	.017**	4.4167	.50361	.041**	4.2917	.69025	1.000
POSTQUES1	3.3750	1.34528		3.7500	1.35935		3.9583	.62409	
PREQUES2	3.9167	.65386	.011**	4.1667	.63702	.026**	4.2500	.44233	.083
POSTQUES2	3.1667	1.16718		3.4583	1.31807		3.5000	.51075	
PREQUES3	4.1250	.61237	.016**	4.2500	.44233	.024**	4.0417	.85867	.046**
POSTQUES3	3.3333	1.27404		3.5417	1.28466		4.2917	.80645	
POSTQUES4	3.4167	.50361	.015**	3.4583	.50898	.038**	4.0833	.50361	.157
POSTQUES4	2.7083	1.19707		2.9583	1.04170		4.0833	.50361	
PREQUES5	4.0417	.85867	.011**	4.0417	.85867	.026**	3.4167	.50361	.083
POSTQUES5	3.1250	1.48361		3.4583	1.17877		4.1667	.76139	

## Discussion

It is broadly believed that the medicinal use of the aromatic odors has an effect on emotional states in humans [14]. Aromatherapy gained attention during the 1980s when work on mind-body healing stimulated the focus on the usage of aromatherapy to alleviate emotional and mental distress. The dental waiting room which is considered a highly anxious place for patients [15].

It is believed that lavender oil acts postsynaptically, and it is suggested that it modulates the activity of cyclic adenosine monophosphate (cAMP). A reduction in cAMP activity is associated with sedation. Linalool which is one of the main ingredients of Lavender oil lowered physiological arousal level by means of autonomic deactivation without affecting mood ratings [7].

Our randomized controlled trial was conducted among orthodontic patients of age 15 to 30 years. The patients in the waiting room were exposed to Lavender and rose oil aromatherapy and compared with the control group. The anxiety was measured by both objective and subjective methods. We found that Lavender oil and Rose oil aromatherapy was significant in the reduction of dental anxiety.

Anxiety scores significantly decreased for the lavender oil group, followed by the rose oil group and were unchanged in the control group. The study findings were in agreement with those from studies conducted by Akbay Oba et al. and Edwards and Fillingim where Lavender oil significantly reduced the anxiety [16,17].

The aroma of essential oils is effective in the reduction of anxiety. The result of our study aligns with the study by Burnett et al., where Rosemary and Lavender scents were used among 73 healthy college students during anxiety-provoking tasks. The previous proved that they significantly reduce tension-anxiety and fatigue-inertia ratings [14]. The result of our study was supported by the results reported by Zabirunnisa et al. which showed a significant reduction in anxiety scores of Lavender group compared with the control group among dental patients above 18 years of age [18].

In our study results (significant reduction of dental anxiety) align with the results reported by Itai et al. where the effects of Lavender and Hiba oil on depression and anxiety among hemodialysis patients was compared [19]. The result of the previous study showed that Hiba oil significantly decreased mean scores on both anxiety and depression and Lavender oil significantly decreased mean anxiety scores.

This study is the first of its kind to compare the effect of lavender oil and rose oil aromatherapy on dental anxiety. It provides evidence for the use of lavender and rose oil aromatherapy in dental settings as a potentially cost-effective intervention for alleviating dental patient anxiety and supports the ancient use of essential oils in the alteration of emotional states. Lavender and rose are an effective means of reducing current anxiety levels and should be perceived as a means of on‑the‑spot reduction of patient anxiety in a dental setting.

The study was conducted on a small sample; future work should consider including a control odor condition when replicating this study on a larger scale.

## Conclusions

Lavender oil and rose oil were efficient in decreasing the anxiety level of patients. Lavender oil had better efficacy in reducing the anxiety level when compared to rose oil. Thus, aromatherapy was efficacious in reducing dental anxiety. Aromatherapy could be considered a complementary, safe therapeutic intervention for the reduction of dental anxiety.
